# Adrenergic Receptor Polymorphism and Maximal Exercise Capacity after Orthotopic Heart Transplantation

**DOI:** 10.1371/journal.pone.0163475

**Published:** 2016-09-26

**Authors:** Mélanie Métrich, Fortesa Mehmeti, Helene Feliciano, David Martin, Julien Regamey, Piergiorgio Tozzi, Philippe Meyer, Roger Hullin, Swiss Transplant Cohort Study

**Affiliations:** 1 Cardiology, Cardiovascular Department, Lausanne University Hospital, University of Lausanne, Lausanne, Switzerland; 2 Cardiology, University Hospital Geneva, University of Geneva, Geneva, Switzerland; 3 Cardiac Surgery, Cardiovascular Department, Lausanne University Hospital, University of Lausanne, Lausanne, Switzerland; 4 Department of Radiology, Lausanne University Hospital, University of Lausanne, Lausanne, Switzerland; Scuola Superiore Sant'Anna, ITALY

## Abstract

**Background:**

Maximal exercise capacity after heart transplantion (HTx) is reduced to the 50–70% level of healthy controls when assessed by cardiopulmonary exercise testing (CPET) despite of normal left ventricular function of the donor heart. This study investigates the role of donor heart β_1_ and β_2_- adrenergic receptor (AR) polymorphisms for maximal exercise capacity after orthotopic HTx.

**Methods:**

CPET measured peak VO_2_ as outcome parameter for maximal exercise in HTx recipients ≥9 months and ≤4 years post-transplant (n = 41; mean peak VO_2_: 57±15% of predicted value). Donor hearts were genotyped for polymorphisms of the β_1_-AR (Ser49Gly, Arg389Gly) and the β_2_-AR (Arg16Gly, Gln27Glu). Circumferential shortening of the left ventricle was measured using magnetic resonance based CSPAMM tagging.

**Results:**

Peak VO_2_ was higher in donor hearts expressing the β_1_-Ser49Ser alleles when compared with β_1_-Gly49 carriers (60±15% vs. 47±10% of the predicted value; *p* = 0.015), and by trend in cardiac allografts with the β_1_-AR Gly389Gly vs. β_1_-Arg389 (61±15% vs. 54±14%, *p* = 0.093). Peak VO2 was highest for the haplotype Ser49Ser-Gly389, and decreased progressively for Ser49Ser-Arg389Arg > 49Gly-389Gly > 49Gly-Arg389Arg (adjusted R^2^ = 0.56, *p* = 0.003). Peak VO_2_ was not different for the tested β_2_-AR polymorphisms. Independent predictors of peak VO_2_ (adjusted R^2^ = 0.55) were β_1_-AR Ser49Gly SNP (*p* = 0.005), heart rate increase (*p* = 0.016), and peak systolic blood pressure (*p* = 0.031). Left ventricular (LV) motion kinetics as measured by cardiac MRI CSPAMM tagging at rest was not different between carriers and non-carriers of the β_1_-AR Gly49allele.

**Conclusion:**

Similar LV cardiac motion kinetics at rest in donor hearts carrying either β_1_-AR Gly49 or β_1_-Ser49Ser variant suggests exercise-induced desensitization and down-regulation of the β_1_-AR Gly49 variant as relevant pathomechanism for reduced peak VO_2_ in β_1_-AR Gly49 carriers.

## Introduction

Cardiopulmonary exercise testing after orthotopic heart transplantation (HTx) shows that maximal exercise capacity is reduced to the 50–70% level of age-matched healthy controls despite of normal left ventricular (LV) ejection fraction [1]. Recipient age, peak heart rate and blood pressure, pulmonary artery resistance, diastolic LV function, BMI, and transplant vasculopathy explain altogether 51–66% of peak VO_2_ variance after transplant [2, 3] suggesting the relevance of other parameters.

Human cardiomyocytes predominantly express the β_1_- and β_2_-adrenergic receptor (AR) subtypes [4] which play a pivotal role for exercise-induced increase in cardiac function. In the healthy heart, the polymorphisms β_1_-AR Ser49Gly, β_1_-AR Arg389Gly, β_2_-AR Arg16Gly, β_2_-AR Gln27Glu, and β_2_-AR Thr164Ile show distinct cardiovascular responses to sympathetic activation [5–8]. However, aortic anastomosis disrupts postganglionic sympathetic innervation and fine-tuned modulation of β-AR activation at the level of the postganglionic nerve-cardiomyocyte synapsis. Consequently, adrenergic stimulation of donor heart function depends on the circulating catecholamine levels [9]. Postganglionic sympathetic fibers of the healthy heart extract large amounts of catecholamines from the circulation [9]. After HTx, postganglionic fibers degenerate, which results in reduced catecholamine retention of the donor heart [10, 11] and 3–5 fold increased circulatory catecholamine levels [12, 13]. We hypothesized that this unique clinical setting in the HTx recipient may change the characteristics of β-AR variant response to adrenergic stimulation because of the different sensitivity of the individual β-AR variant to downregulation when exposed to increased catecholamine concentration.

The outcome parameter of this correlational study was peak VO_2_ measured by cardiopulmonary exercise testing. Peak heart rate and systolic blood pressure are variables that determine maximal exercise capacity after HTx [2, 3]. Both variables depend on circulating catecholamines, therefore, all study patients were screened for recipient expression of α_2c_-AR Del 322–325 variant, which strengthens activation of the adrenal gland chromaffin cell [14] with subsequent increased spillover of norepinephrine and epinephrine into the circulation [15].

## Methods

### Study design and population

This study complies with the Declaration of Helsinki and was approved by the ethical committee of the canton de Vaud. Written informed consent was obtained from all patients. This is a substudy of the prospective epidemiological cohort study following solid organ transplant recipients in Switzerland (Swiss Transplant Cohort Study, STCS No.0038). Inclusion criteria were stable adult HT recipients ≥9 months and <4 years post-transplant with a maximal cardiopulmonary exercise test (CPET) as defined by the achievement of a respiratory exchange ratio (RER) >1.1 [16]. Exclusion criteria were age: 1. <18 years; 2. missing consent; 3. ≥moderate severity of comorbidity limiting execution of maximal CEPT; 4. presence of ≥moderate acute allograft rejection (International Society for Heart and Lung Transplantation grade ≥2R) [17] in the endomyocardial biopsy; 5. ≥moderate cardiac allograft vasculopathy in the coronary angiogram at the time of exercise testing; or 6. severe valvular dysfunction (insufficiency ≥III/IV or more than ≥moderate stenosis) as assessed by standard echocardiography performed by a board-certified cardiologist <1 month before CPET.

### Demographic and clinical data

Demographic and clinical data at CPET were collected from electronic charts. Standard laboratory tests included sodium, potassium, creatinine, hemoglobin, and brain natriuretic peptide (BNP) level.

### Maximal cardiopulmonary exercise testing

All patients underwent maximal CPET. Patients were tested using an electrically braked cycloergometer (Ergoline 900/911 digital, Ergoline GmbH, Germany) with individualized ramp protocol. Respiratory gas exchange was measured breath-by-breath with determination of peak VO_2_, VO_2_AT (VO_2_ at anaerobic threshold), VE/VCO_2_ (ventilatory efficiency), ventilatory reserve and RER. Maximal exercise capacity was determined by the highest VO_2_ achieved during the last 30 seconds of maximal exercise. Heart rate, blood pressure, and 12-lead ECG were continuously recorded throughout exercise and recovery.

### Circumferential strain measurement of the donor heart

Patients underwent cardiovascular magnetic resonance imaging in order to study the influence of the β_1_−49 genotype on LV function. All patients were scanned with a prototype slice followed balanced Steady State Free Precession (bSSFP) CSPAMM (Complementary Spatial Modulation of Magnetization) tagging technique [18, 19]. All scans were performed on a clinical MAGNETOM Verio 3T scanner (Siemens Healthcare, Erlangen, Germany). Scout scans were acquired to find the short axis (SA) of the LV and place the tagged slices: for each exam, three SA slices were acquired at basal, mid-ventricular and apical levels of the LV. The basal slice was acquired 1 cm below the mitral valve, the apical slice 1 cm above the endomyocardial border of the apex, and the mid-ventricular slice was centered between the two other slices. The imaging parameters for the SF bSSFP CSPAMM sequence were as follows: TE/TR = 1.38/3.2ms, 12° radio frequency excitation angle, a bandwidth of 849 Hz/pixel, a temporal resolution of 45 ms, (82–85)*256 matrix size, with 32 to 33% of phase resolution. The tagged slice thickness was 6 mm, while the imaged slice thickness was 20 mm, 25 and 30 mm, respectively, at apex, mid-ventricle and base in order to keep the tagged slice in the imaged slab at all times. Each slice was acquired in a 16 heartbeats breath hold. All tagged images were analyzed using Harmonic Phase Imaging (HARP) [20], in the Virtue software from Diagnosoft (HARP, v4.1, Diagnosoft Inc., Palo Alto, CA, USA). Circumferential strain measurements could be obtained from the analysis as an average over each slice. All obtained values were adapted to the duration of the systole [21] using a custom Matlab script (The Mathworks, Inc, Natick, MA, USA), in order to create a meaningful comparison. For comparison of circumferential strain measurements between the two β_1_−49 genotypes groups (see above) unpaired Student’s t-tests corrected for samples of different sizes were used.

### Polymorphism genotyping

Donor genomic DNA was extracted from paraffin-embedded endomyocardial biopsy specimen using Purelink genomic DNA kit (Invitrogen®). Extracted leucocyte DNA provided by the STCS biobank was used for recipient genomic DNA analysis. Detection of AR polymorphism used Taq-Man single-nucleotide polymorphism genotyping assays (Life Technologies®). SNP ID were: rs1801252 for β_1_-AR Ser49Gly, rs1801253 for β_1_-AR Arg389Gly, rs1042713 for β_2_-AR Arg16Gly, rs1042714 for β_2_-AR Gln27Glu and rs1800888 for β_2_-AR Thr164Ile. Genotype assignments were obtained by fluorescence measurement using an ABI Prism 7500 Sequence Detection System with its allelic discrimination software (Life Technologies®).

α_2C_-AR polymorphism was examined after amplification of recipient genomic DNA by PCR. Primers for PCR were: 5’-ACGTGGAGCCGGACGAGA-3’ (sense) and 5’-GTTCTTCCTGTCGCGCCG-3’ (antisense). The PCR consisted of 5 ng of genomic DNA, 1 pmol of each primer, 0.2 mM dNTPs, 1 unit of Gotaq DNA polymerase (Promega®), 4 μl of 5X GoTaq buffer and 5% DMSO in a 20 μl reaction volume. Reactions were started by an initial incubation at 94°C for 4 min, followed by 35 cycles of 94°C for 30 s, 65°C for 30 s, and 72°C for 30 s, followed by a final extension at 72°C for 10 min. PCR products were digested with HaeIII (Life Technologies®) at 37°C for one hour since α_2C_ Del322-325 results in the loss of one of four HaeIII restriction sites in α_2C_.

### Statistical analysis

Allele frequencies were computed by standard gene-counting methods. Association of peak VO_2_ with demographic and clinical parameters, or AR SNPs was tested using univariate regression analysis with peak VO_2_ as the dependent variable. Peak VO_2_ was expressed as the percentage of the predicted value (% predicted), which is already adjusted for age, BMI and gender [22]. Thus, these three co-variables were not included in the univariate and multivariate analysis. β_1_−49 genotype was considered as Gly carriers when homozygous or heterozygous, in accordance with the literature [5, 23]. The mean peak VO_2_ of each AR genotype suggested a dominant and a recessive model for the β_1_−389 and the β_2_ genes, respectively. Thus, β_1_−389 genotype was considered as homozygous Arg389Arg or Gly389 carriers; β_2_ genotype was considered homozygous Arg16Arg or Gly16 carriers and homozygous Glu27Glu or Gln27 carriers; and α_2C_ genotype was considered as homozygous WT or carriers of the deletion 322–325. Because of the low number of patients carrying the β_2_ Ile164 variant (n = 2/41), this SNP did not enter into the final analysis.

Secondary clinical variables included continuous and categorical clinical variables. Categorical variables were defined as non-treated or treated by a certain drug. Cut-off for BNP and N-terminal propeptide BNP (NT-proBNP) levels were 100 ng/l and 300 ng/l, respectively, in accordance with the heart failure guidelines of the European Society of Cardiology. Continuous clinical data were expressed as mean±S.D.; a *p*-value <0.05 was considered as statistically significant.

Multivariable logistic regression using backward analysis of parameters correlating with *p*<0.10 was performed to estimate the association between explanatory variables and peak VO_2._ Association of a β-AR genotype with peak VO_2_ was considered statistically significant when *p* was <0.0125 since 4 β-AR SNPs entered the final analysis.

## Results

### Patient inclusion

A total of 59 HTx recipients was screened; 18 patients met the following exclusion criteria: consent refusal (n = 2), age <18 years (n = 2), CPET not performed (n = 12), endomyocardial biopsy obtained late after CPET (n = 1), ≥moderate severity of comorbidity (n = 1).

### Patient characteristics

S1 Table shows baseline characteristics of the 41 HTx recipients (6 females). Gender distribution in this present study corresponds the one reported in the registry of the International Society of Heart and Lung transplantation [24]. Mean age at HTx was 51±12 years with a mean time interval of 560±309 days between HTx and CPET. BMI was 25.6±4.6.

Echocardiographic left and right ventricular systolic function indices were in the lower normal range (LVEF: 60±7%; S’ wave 9.7±2.9 cm/s; TAPSE 14.4±4.0 mm). Mean lateral E/E’ ratio was 8.3± 3.0. The mean creatinine level was increased (139±42 μmol/l), the estimated glomerular filtration rate (eGFR) using the CKD-EPI (Chronic Kidney Disease Epidemiology Collaboration) was 60±28 ml/min; 55% of the patients presented an eGFR ≤60 ml/min. Mean hemoglobin level was 125±19 g/l; levels of BNP or NT-proBNP were increased in 49% of study patients.

All patients were on immunosuppressive drugs. Cardiovascular medications at the time of cardiopulmonary exercise testing included diuretics (41%), β-blockers (metoprolol) (32%) and calcium channel blockers (amlodipine) (22%).

No study patient had histological signs of acute rejection in the endomyocardial biopsy obtained before CPET.

### Maximal cardiopulmonary exercise testing

CPET parameters are shown in S2 Table. Mean peak VO_2_ was 17.1±6.2 ml/kg/min corresponding to 57.0±14.8% of the predicted value adjusted for age, BMI and gender; the mean peak power output was 107±51 Watts. VO_2_AT was 12.0±4.1 ml/kg/min corresponding to 41±12% of the predicted value of AT. Ventilatory efficiency (measured by the VE/VCO_2_ slope) was 33.2±5.7; peak ventilatory reserve was 43±16%. The RER was 1.27±0.14 at maximal exercise level.

Resting heart rate (HR) was 93±13 beats per minute (bpm), peak HR was 126±22 bpm, chronotropic reserve (ΔHR) was 33±16 bpm. Chronotropic incompetence, defined as failure to achieve 85% of the age-predicted maximal HR [2], was present in 76% of the patients. HR recovery during the first minute after cessation of exercise was 7±11 bpm (71% presented a value <12 bpm; ≥12 bpm is considered normal). Blood pressure (BP) increased with exercise (rest vs. maximal exercise: systolic BP 121±16 vs. 169±21 mmHg; diastolic BP 80±11 vs. 86±13 mmHg).

### Peak VO_2_ and adrenergic receptor polymorphism

Frequency of β-AR variants in donor hearts of study patients matched with β-AR variant distribution in the European population (Table 1) [8, 25–29].

**Table 1:** Distribution of adrenergic receptor variants among patients. WT = Wild-type; Mut = Mutant; MAF = mean allele frequency; MAF population.

Gene	Amino Acid variation	Patients number			MAF CHUV	MAF
		WT/WT	WT/Mut	Mut/Mut		Population
β_1_-AR	49 (Ser>Gly)	31	9	1	13%	12%
	389 (Arg>Gly)	23	14	4	27%	27%
β_2_-AR	16 (Arg>Gly)	16	19	6	38%	37%
	27 (Gln>Glu)	12	21	8	45%	43%
	164 (Thr>Ile)	39	2	0	2%	1%
α_2C_-AR	Deletion 322–325	37	3	1	6%	4%

**Fig 1:**
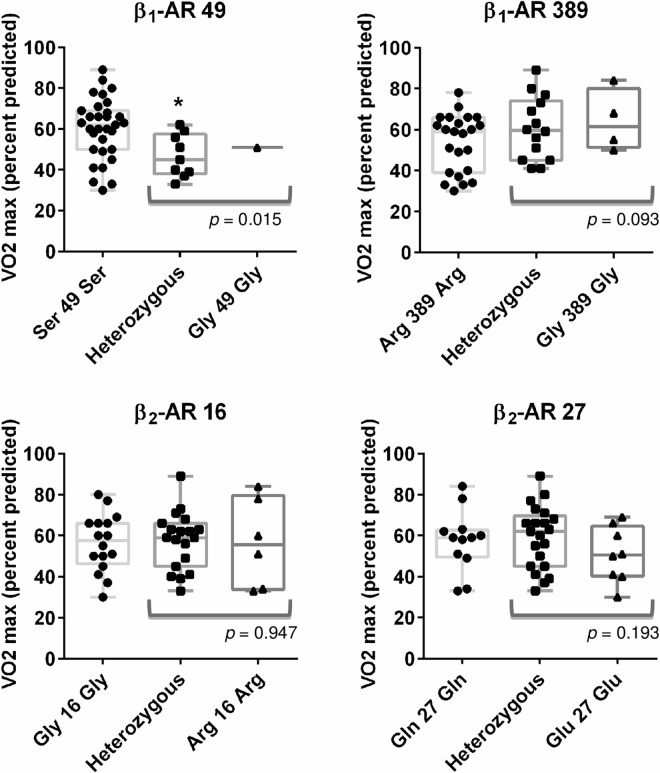
β_1_-AR and β_2_-AR SNPs and maximal exercise capacity. Peak VO_2_ is shown for β_1_-AR codon 49 and 389, β_2_-AR codon 16 and 27. Box graphs represent median, upper/lower quartiles and maximum/minimum values. *indicates a statistically significant difference (p <0.05) between SNP and peak VO_2_. Figures represent box plot for each genotype combination (homozygous for the major allele, WT/WT, heterozygous WT/minor allele and homozygous for minor allele).

**Fig 2:**
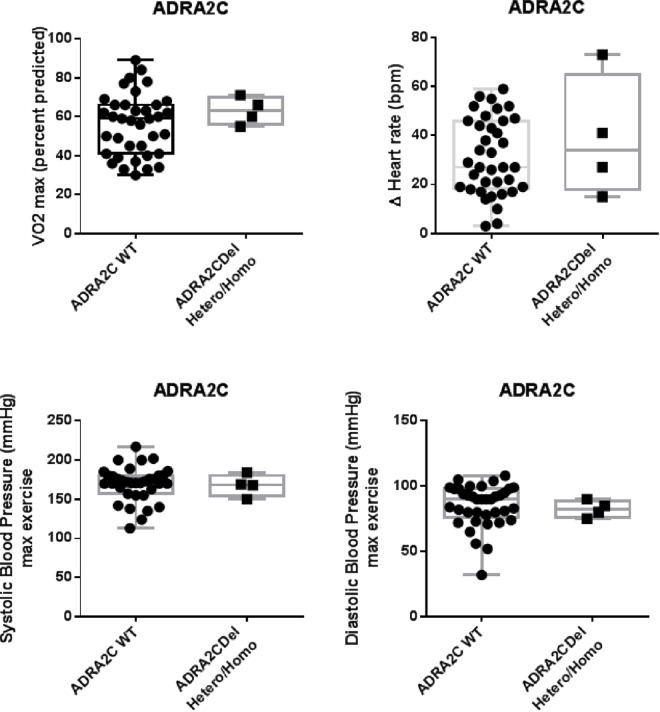
Effect of α_2C_-adrenergic receptor polymorphism on maximal exercise capacity. Cardiopulmonary exercise parameters are presented according to genotype α_2c_-AR (WT or 322-325deletion). Box graphs represent median, upper/lower quartiles and maximum/minimum values. No statistically significant correlation (p <0.05) was found between α_2C_-Del322-325 and variables (peak VO_2_, ΔHR and BP). Hetero/Homo = heterozygous/homozygous

Fig 1 shows peak VO_2_ as a function of β-AR variant expression in the allograft. Patients with grafts carrying the β_1_-AR Gly49 allele had a significantly lower predicted percentage of peak VO_2_ when compared to homozygotes Ser49Ser (47.3±10.0% vs. 60.2±14.9%; *p* = 0.015). β_1_-AR Gly49 carriers had also more chronotropic incompetence than Ser49Ser group even if this was not significant (29±11 vs. 35±18; p = 0.327) (S3 Table). There was a trend towards increased peak VO_2_ with Gly389 carriers when compared to homozygotes Arg389Arg (61.4±14.9 vs. 53.6±14.1%; *p* = 0.093). Polymorphism of β_2_-AR 16 in the donor heart did not affect peak VO_2_ (*p* = 0.947), whereas peak VO_2_ was by trend lower in β_2_-AR Glu27Glu patients when compared to Gln27 carriers (50.9±13.6% vs. 58.5±14.9%; *p* = 0.193). α_2C_ polymorphism did not interact with peak VO_2_ (*p* = 0.347), chronotropic reserve (*p* = 0.729), systolic (*p* = 0.971) or diastolic BP (*p* = 0.967) (Fig 2).

### Correlation of peak VO_2_ with clinical variables

Univariate regression analysis showed correlation of percent predicted peak VO_2_ adjusted for recipient age, BMI and gender with chronotropic reserve (r^2^ = 0.35; *p*<0.001), HR recovery (r^2^ = 0.19; *p* = 0.006), peak systolic blood pressure (BP) (r^2^ = 0.18; *p* = 0.008) and Δsystolic BP (r^2^ = 0.13; *p* = 0.024). Furthermore, peak VO_2_ was reduced with renal dysfunction (eGFR ≤60 ml/min) (r^2^ = 0.27; *p*<0.001), increased BNP or NT-proBNP levels (r^2^ = 0.14; *p* = 0.014), or reduced hemoglobin concentration (r^2^ = 0.14; p = 0.017). Peak VO_2_ was reduced with diuretic therapy (r^2^ = 0.13; p = 0.019) (S4 Table and S1 Fig). None of the included echocardiographic parameters showed a relevant correlation with peak VO_2_.

### Predictors of peak VO_2_

Peak HR as well as Δsystolic BP were considered as variables depending on chronotropic reserve or peak systolic BP respectively, and were thus not included in the final model. Likewise, creatinine was considered as dependent of the eGFR. Multivariable analysis showed that the β_1_-AR Gly49 variant of the donor heart (*p* = 0.005), along with ΔHR (*p* = 0.016) and peak systolic BP (*p* = 0.031) were independently associated with peak VO_2_ (adjusted R^2^ = 0.55) (Table 2). The β_1_-AR Gly49 polymorphism remained correlated to peak VO_2_ when β-blocker treatment was forced into the model.

**Table 2:** Multivariable correlation between peak VO_2_, β_1_-AR genotype and clinical variables. ΔHR = Peak heart rate—resting heart rate; BP = Blood Pressure; eGFR = estimated glomerular filtration rate.

Principal variable : peak VO_2_ (% predicted)	
Number of observations : 39	
R^2^: 0.61	
Adjusted R^2^ : 0.55	
Co-variables	P value
β_1_ Gly49	0.005
Exercise modality	0.108
ΔHR	0.016
Peak systolic BP	0.031
eGFR	0.187

### Haplotype and peak VO_2_

**Fig 3:**
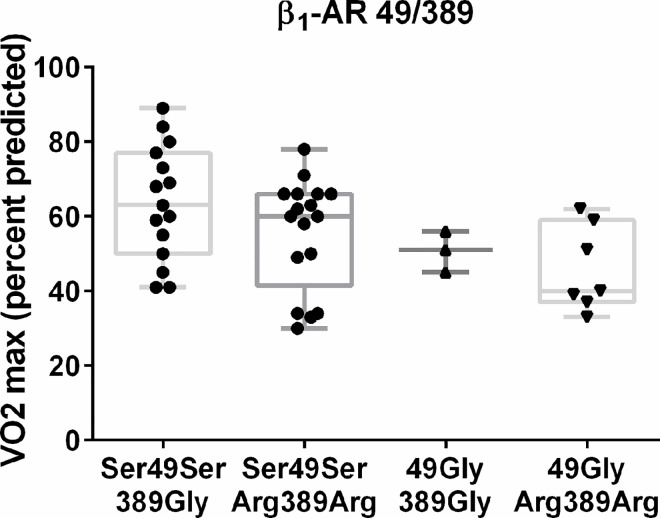
β_1_-AR 49 and β_1_-AR 389 haplotypes and peak VO_2._ Maximal exercise capacity was assessed by measuring peak VO_2_ and was expressed according to the patient haplotypes β_1_-49Gly+β_1_-Arg389Arg, β_1_-49Gly+β_1_-389Gly, β_1_-Ser49Ser+β_1_-Arg389Arg or β_1_-Ser49Ser+β_1_-389Gly. Box graphs represent median, upper/lower quartiles and maximum/minimum values.

Peak VO_2_ was not different by ANOVA between the haplotypes of β_1_-49Gly+β_1_-Arg389Arg, β_1_-49Gly+β_1_-389Gly, β_1_-Ser49Ser+β_1_-Arg389Arg, β_1_-Ser49Ser+β_1_-389Gly. Nevertheless, peak VO_2_ correlated with the different haplotypes fitting to a linear regression (adjusted R^2^ = 0.17; p = 0.005) with lowest mean peak VO_2_ values for β_1_-49Gly+β_1_-Arg389Arg and β_1_-49Gly+β_1_-389Gly (Fig 3). This correlation remained consistent (adjusted R^2^ = 0.56; *p* = 0.003) when adjusted for other modalities affecting peak VO_2_ (ΔHR, maximal systolic BP, eGFR).

### Circumferential strain measurement by tagging

**Fig 4:**
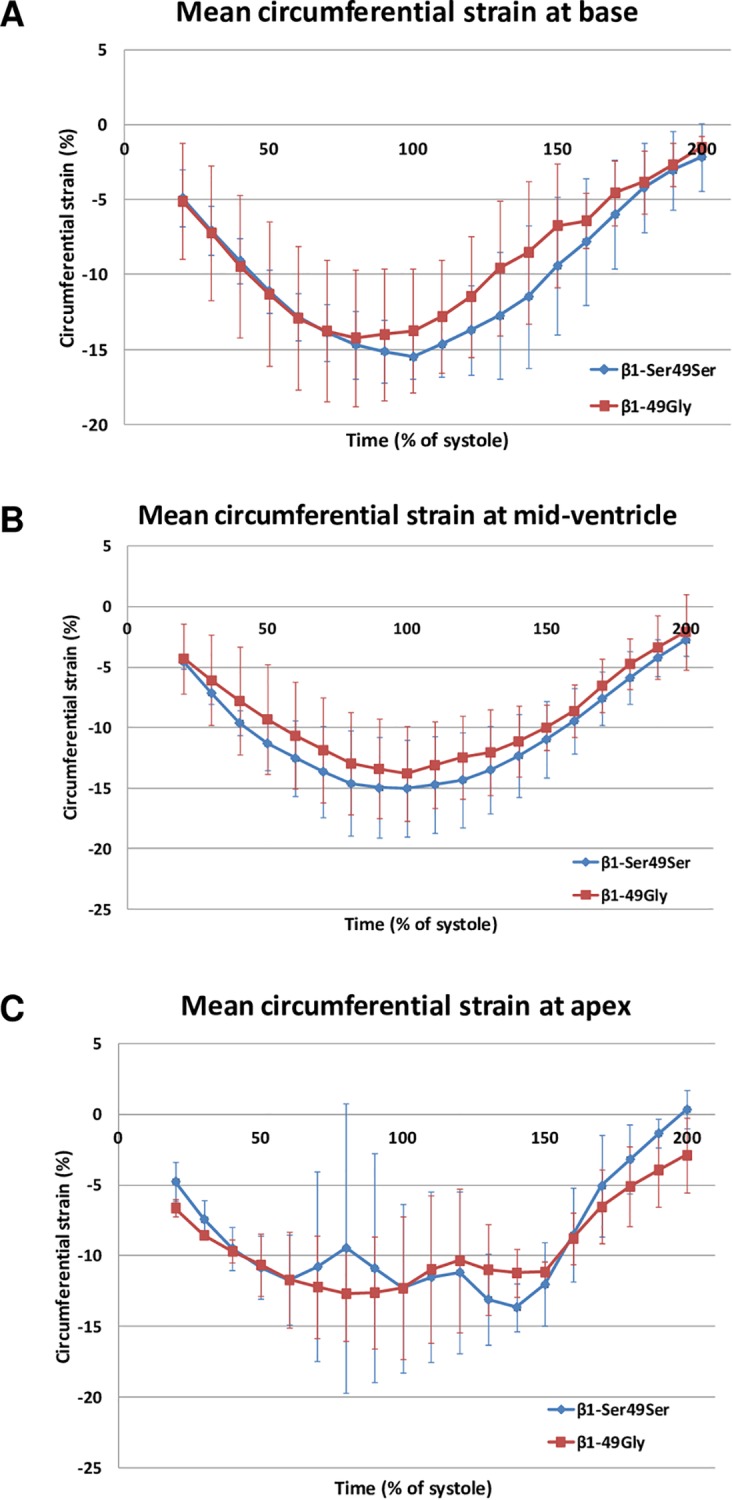
Mean circumferential strain at basal, mid-ventricular and apical slices of the left ventricle comparing β_1_-AR Ser49Ser and β_1_-Gly49 carriers along the cardiac cycle. The strain values were obtained using HARP analysis on tagged images acquired with a SF bSSFP CSPAMM tagging technique. All measurements were adapted to the systole duration of each exam. No statistically significant difference could be found between the two groups.

Nine patients (3 women; age 46±14 y) were scanned. Circumferential strain measurements were obtained as described above; one slice with artifact was not included in the analysis (n = 1/27 slices). Patients were grouped into carriers of the β_1_-AR Gly49 variant (n = 4) and patients with the β_1_-AR Ser49Ser variant (n = 5). Groups were without significant difference at any time of the cardiac cycle as assessed at the time point of largest difference (Fig 4A base: -12.7±4.2 vs. -9.6±4.5% at 130% of systole duration, *p* = 0.32; Fig 4B mid-ventricular: -14.6±4.3 vs. -13.0±4.2% at 80% of systole duration, *p* = 0.60; Fig 4C apical: -9.4±10.2% vs. -12.7±3.4% at 80% of systole duration, *p* = 0.58).

## Discussion

By showing that β-adrenergic receptor polymorphism in the donor heart is a determinant of peak VO_2_, the present study adds to the pathophysiological understanding of limited maximal exercise capacity in HTx recipients with normal left ventricular function at rest.

### Study population

The hypothesis that polymorphism of the donor heart β-AR or the recipient α_2c_-AR affects maximal exercise capacity after orthotopic HTx was tested in a prospective cohort study design including consecutive patients. HTx recipients were not included when transplanted less than 9 months ago because maximal exercise capacity increases after transplantation reaching a plateau only after the first 9 postoperative months [2, 30–32]. In addition, patients were included only when <transplanted less than 4 years ago acknowledging the observation of functional donor heart reinnervation in a very small minority of HTx recipients >4 years post-transplant [33]. Because of the applied selection criteria, characteristics of the present study population might differ from the patient profile reported in other exercise studies after HTx [3, 15, 23, 30–32, 34]. However, recipient age, gender distribution, time interval after HTx, BMI, and left ventricular ejection fraction at rest compare to other reports [3, 23, 30–32, 34]. Furthermore, peak VO_2_ adjusted for age, BMI, and gender as well as hemoglobin and creatinine levels are similar to characteristics reported for HTx recipients >1 year post-transplant in other exercise studies [30, 32]. However, in the present study donor age was higher (41 vs. 26–30 years) and chronotropic reserve was lower (33 vs. 37–47 bpm) [2, 3, 30, 32, 35]. In theory, the low-dose metoprolol treatment (in the present study: mean dose 24±10 mg/d) administered in almost one third of the study participants at the moment of CPET might decrease chronotropic reserve. However, multivariable analysis did not show interaction between β-blocker treatment and peak VO_2_, and, chronotropic reserve remained predictive for peak VO_2_ even when β-blocker treatment was forced into the predictive model. Furthermore, Doesch et al. reported unchanged peak VO_2_ in their exercise study, which assessed HTx recipients before and during high β-blocker treatment (mean metoprolol dose: 147±53 mg/d) [36]. Altogether, this suggests that the lower chronotropic reserve in the present study should relate to elder donor age as reported elsewhere [34].

### Adrenergic receptor polymorphism and orthotopic heart transplantation

In this study, the deletion variation of the α_2c_-adrenoceptor did not interact with peak VO_2_, chronotropic reserve, systolic or diastolic BP suggesting that the minor allele is not associated with a distinct exercise-associated phenotype in HTx recipients. This interpretation is in accordance with the absence of a distinct phenotype of the minor allele in populations with other cardiovascular disease [5] but has to consider the small number of study participants carrying the deletion variant. However, the proportion of study patients with the minor allele compares to the allele frequency reported in other populations [26, 27].

Frequencies of the donor heart β_1_- or β_2_-AR alleles correspond to respective reports from the California Donor Transplant Network [37] and the European population [27–29] suggesting the absence of a selection bias in the present cohort. Previous studies have shown that β-adrenergic signal transduction in the cardiac allograft is altered as a consequence of a decreased G_Sα_ expression [38] but this change does not explain the individual variation of maximal exercise capacity after HTx. Sustained agonist exposure may differently affect β-AR variant desensitization and down-regulation [39]. Therefore, we hypothesized that increased circulatory catecholamine levels in combination with the specific biochemical characteristics of the tested β-AR variants should explain interindividual variation of maximal exercise capacity after HTx. Especially, the β_1_-49Gly and the β_1_-Arg389 variants exhibit greater agonist-promoted desensitization when exposed to saturating catecholamine concentrations while the β_1_-Ser49 variant is resistant to agonist-promoted downregulation [25, 40, 41]. In concordance, the haplotype β_1_-Gly49/Arg389Arg shows more rapid agonist-promoted receptor down-regulation and desensitization in vitro when compared with other haplotypes [42]. In the present study, the β_1_-Ser49Ser variant was associated with significantly higher peak VO_2_ values when compared with the β_1_-AR Gly49 variant while there was no significant correlation with the β_1_-AR Arg389 or the various β_2_-AR variants tested. Moreover, the donor heart Ser49Ser+Gly389Gly haplotype was related with the highest peak VO_2_ levels while peak VO_2_ decreased progressively for the haplotypes Ser49Ser+Arg389Arg > 49Gly+389Gly > 49Gly+Arg389Arg. We thus identified β_1_-AR polymorphism at position 49 and the haplotype combination of β_1_-AR49 + β_1_-AR389 polymorphisms as independent predictors of exercise performance after orthotopic HTx.

Studies in patients with coronary artery disease or heart failure, however, have shown higher peak VO_2_ values in carriers of either β_1_-AR 49Gly or β_1_-AR 389Arg variant, or the haplotype 49Gly/389Arg [43, 44]. The appraisal of these contrasting results has to consider the 3–5 fold increased level of circulatory catecholamines in HTx recipients at rest, which increases even further with exercise. In the present study, baseline VO_2_, circumferential cardiac fiber shortening kinetics at rest, and chronotropic reserve were not different between carriers of the β_1_-AR Gly49 and β_1_-AR Ser49 variant suggesting that the lower peak VO_2_ in β_1_-AR 49Gly carriers should relate to a relative decrease of myocardial contractility at peak exercise. This conclusion is compatible with the substantial down-regulation of β_1_-AR 49Gly membrane expression shown for cells exposed to high catecholamine levels (27). The β_1_-AR Ser49 variant, however, seems to maintain agonist promoted stimulation of myocardial contractility during peak in the HTx recipient because of its resistance to agonist-promoted downregulation (27). Finally, physiological studies in HTx recipients have shown the important role of the β_2_-AR for heart rate (36, 37), which can explain why β_1_-AR Gly49Ser variants impact on myocardial contractility at peak exercise without affecting peak heart rate.

### Limitations of the study

This study collective includes only a small number of patients. However, demographic and clinical characteristics of the study population as well as distribution of allele frequencies are in accordance with reports from other populations. Despite of the fact that this study provides solid indirect evidence suggesting that exercise induces desensitization and down-regulation of the β_1_-AR Gly49 variant, direct proof is missing. However, proof of β-AR down-regulation with peak exercise *in vivo* or measurement of circumferential shortening at maximal exercise in the cardiac MRI is not feasible. Quantification of circumferential cardiac kinetics by echocardiography may be an alternative but may miss differences due to larger standard deviation.

## Conclusion

Reduced exercise capacity remains a concern after HTx because more recent advance in immunosuppression permits long survival, which is why quality of life aspects such as daily physical activity gain importance. This study demonstrates that maximal exercise capacity as measured by peak VO_2_ is reduced in HTx recipients carrying a donor heart expressing the β_1_-AR 49Gly variant. The results of the present study suggest that HTx recipients carrying this polymorphism in their donor heart should benefit from high-dose β_1_-AR blockade.

## Supporting Information

S1 Fig Exercise or clinical variables significantly affecting peak VO_2._ Changes in peak VO_2_ was plotted according to exercise modality, BNP or NT-proBNP levels, treatment by diuretics, peak HR, chronotropic reserve (ΔHR) or HR recovery at 1 min, peak or Δsystolic BP (peak or delta systolic BP), hemoglobin levels, creatinine blood levels and the glomerular filtration rate estimated (eGFR) using the CKD-EPI formula adjusted for weight. Box graphs represent median, upper/lower quartiles and maximum/minimum values. Mean ± S.D of peak VO_2_ with *r*^*2*^ and *p* value are shown on top of each box. Linear regression curves are represented in blue.
(TIF)

S1 Table Patient baseline clinical variables. LVEF = left ventricular ejection fraction, LVMI = left ventricular mass index, TAPSE = tricuspid annular plane systolic excursion, eGFR = estimated glomerular filtration rate; BNP = brain natriuretic peptide; NT-proBNP = N-terminal pro-brain natriuretic peptide; ACE-I = angiotensin converting enzyme-inhibitor; ARB = angiotensin II receptor blocker type 1.
(TIF)

S2 Table Cardiopulmonary exercise parameters. AT = anaerobic threshold; RER = respiratory exchange ratio; HR = heart rate; BP = blood pressure; ΔBP = Peak BP- resting BP
(TIF)

S3 Table Comparison of clinical variables between β1 49Gly carriers and β1 Ser49Ser group. LVEF = left ventricular ejection fraction, LVMI = left ventricular mass index, TAPSE = tricuspid annular plane systolic excursion, eGFR = estimated glomerular filtration rate; BNP = brain natriuretic peptide; NT-proBNP = N-terminal pro-brain natriuretic peptide; ACE-I = angiotensin converting enzyme-inhibitor; ARB = angiotensin II receptor blocker type 1. AT = anaerobic threshold; RER = respiratory exchange ratio; HR = heart rate; BP = blood pressure.
(TIF)

S4 Table Univariate analysis showing the correlation between peak VO_2_ and clinical or exercise variables. LVEF **=** left ventricular ejection fraction, LVMI **=** left ventricular mass index, TAPSE **=** tricuspid annular plane systolic excursion; HR **=** heart rate; ΔHR **=** Peak HR—resting HR; BP **=** Blood Pressure; ΔBP **=** Peak BP- resting BP; eGFR **=** estimated glomerular filtration rate; BNP **=** brain natriuretic peptide; NT-proBNP **=** N-terminal pro-brain natriuretic peptide; ACE-I **=** angiotensin converting enzyme-inhibitor; ARB **=** angiotensin II receptor blocker type 1.
(TIF)
