# Supplementary figures and images for: Adrenergic Receptor Polymorphism and Maximal Exercise Capacity after Orthotopic Heart Transplantation
Source: PLoS One. 2016 Sep 26;11(9):e0163475. doi: 10.1371/journal.pone.0163475 (PMC5036840; doi:10.1371/journal.pone.0163475)

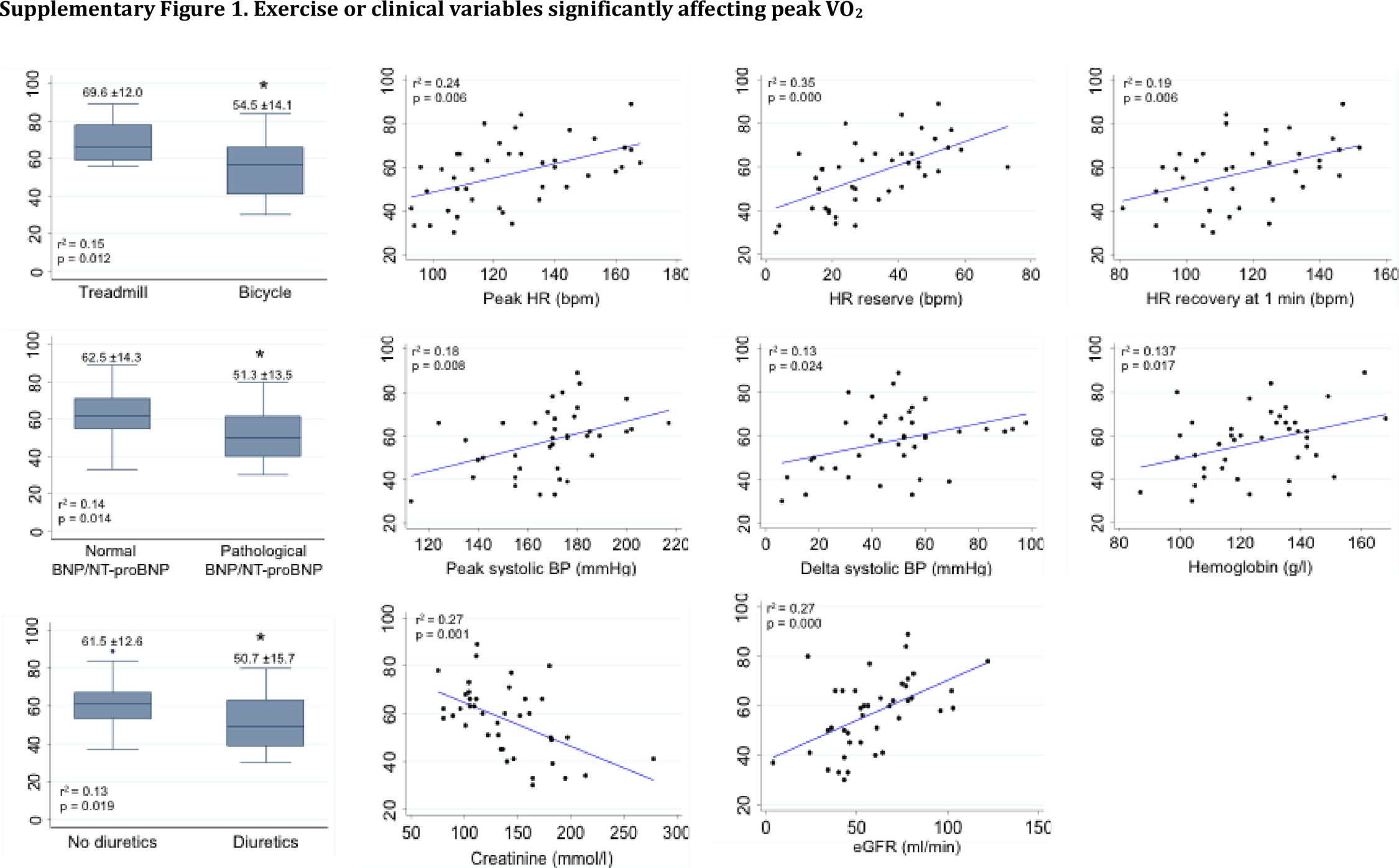

Supplement: S1 Fig — Changes in peak VO2 was plotted according to exercise modality, BNP or NT-proBNP levels, treatment by diuretics, peak HR, chronotropic reserve (ΔHR) or HR recovery at 1 min, peak or Δsystolic BP (peak or delta systolic BP), hemoglobin levels, creatinine blood levels and the glomerular filtration rate estimated (eGFR) using the CKD-EPI formula adjusted for weight. Box graphs represent median, upper/lower quartiles and maximum/minimum values. Mean ± S.D of peak VO2 with r2 and p value are shown on top of each box. Linear regression curves are represented in blue. (TIF) [file pone.0163475.s001.tif]

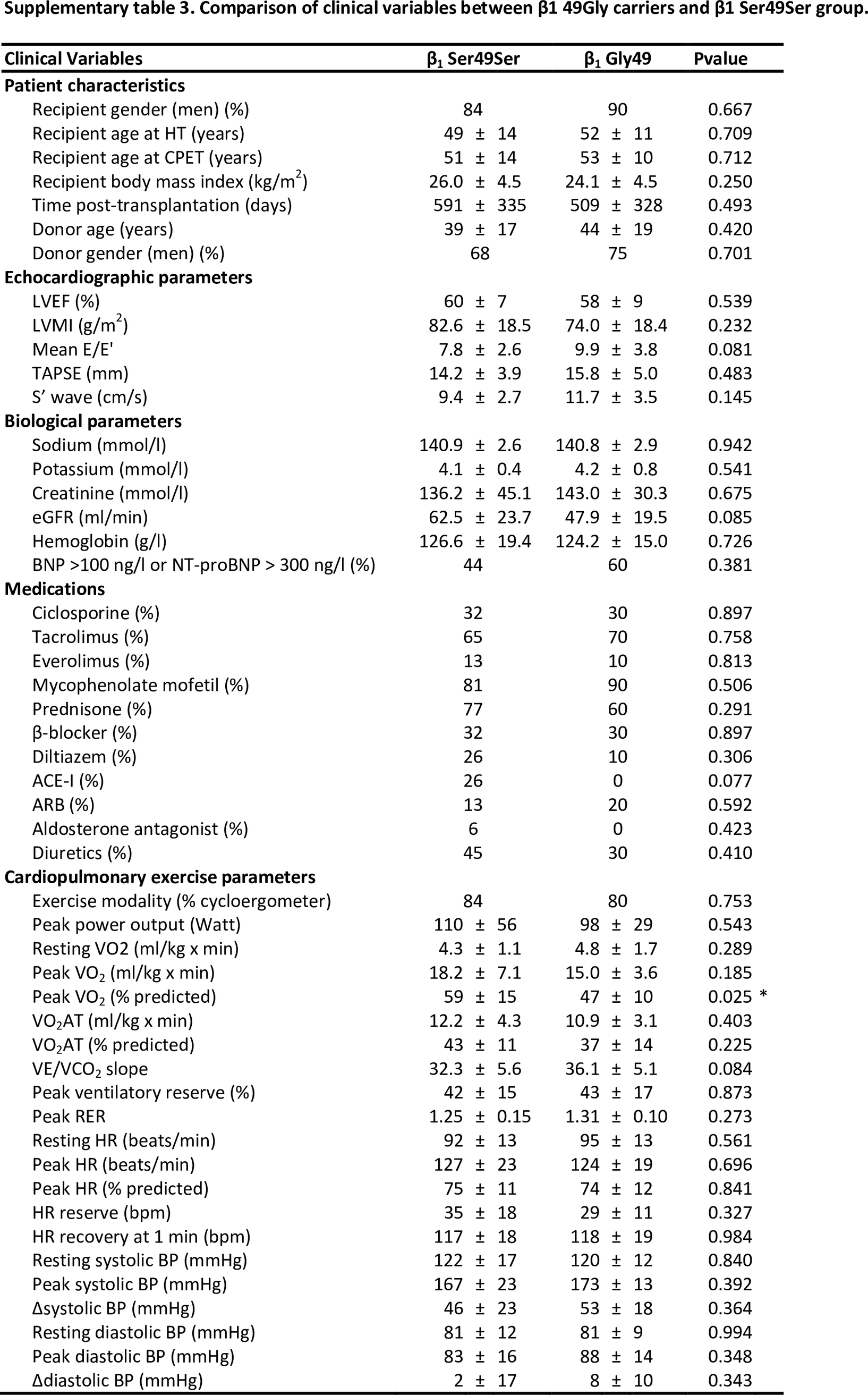

Supplement: S1 Table — LVEF = left ventricular ejection fraction, LVMI = left ventricular mass index, TAPSE = tricuspid annular plane systolic excursion, eGFR = estimated glomerular filtration rate; BNP = brain natriuretic peptide; NT-proBNP = N-terminal pro-brain natriuretic peptide; ACE-I = angiotensin converting enzyme-inhibitor; ARB = angiotensin II receptor blocker type 1. (TIF) [file pone.0163475.s002.tif]

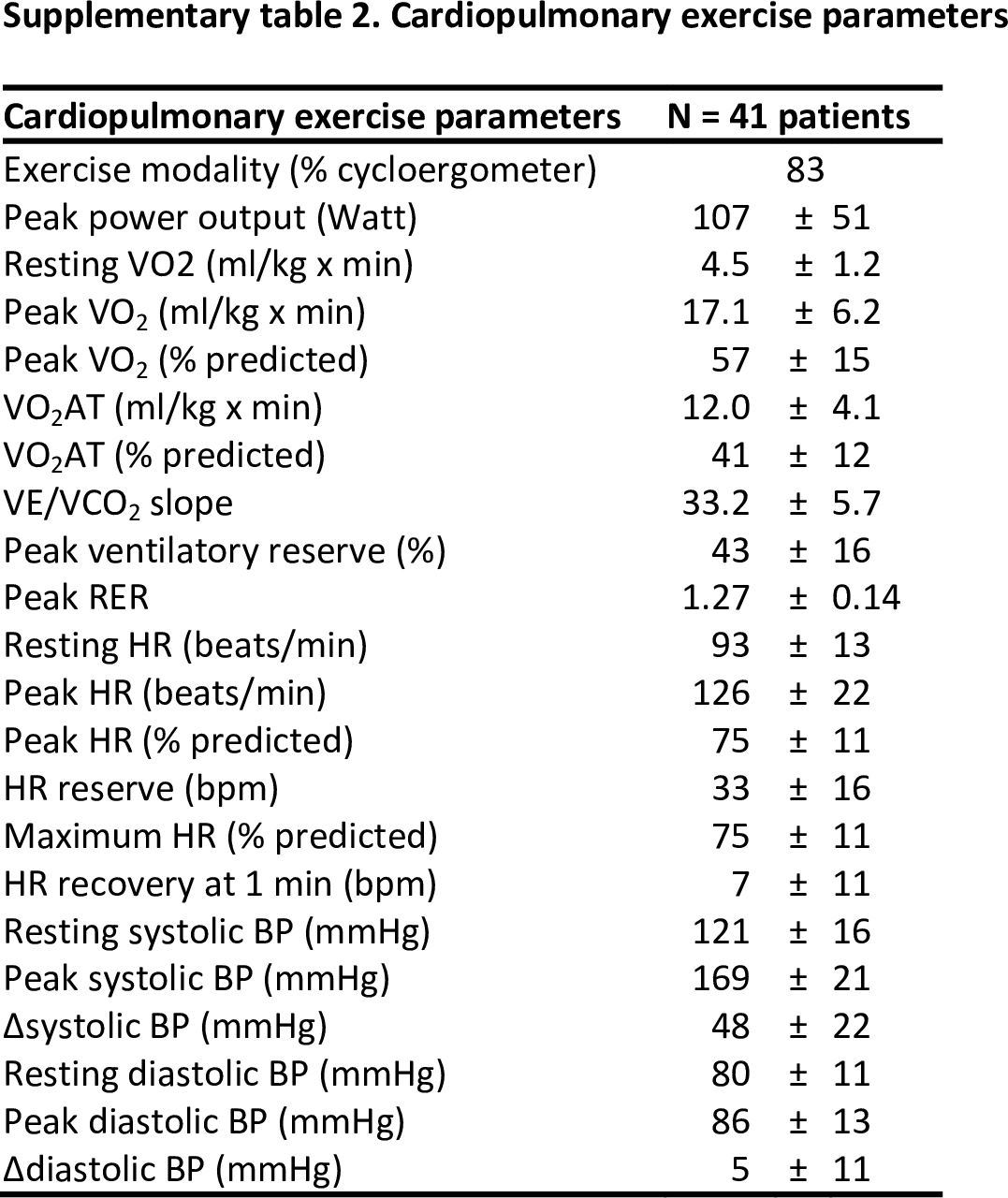

Supplement: S2 Table — AT = anaerobic threshold; RER = respiratory exchange ratio; HR = heart rate; BP = blood pressure; ΔBP = Peak BP- resting BP (TIF) [file pone.0163475.s003.tif]

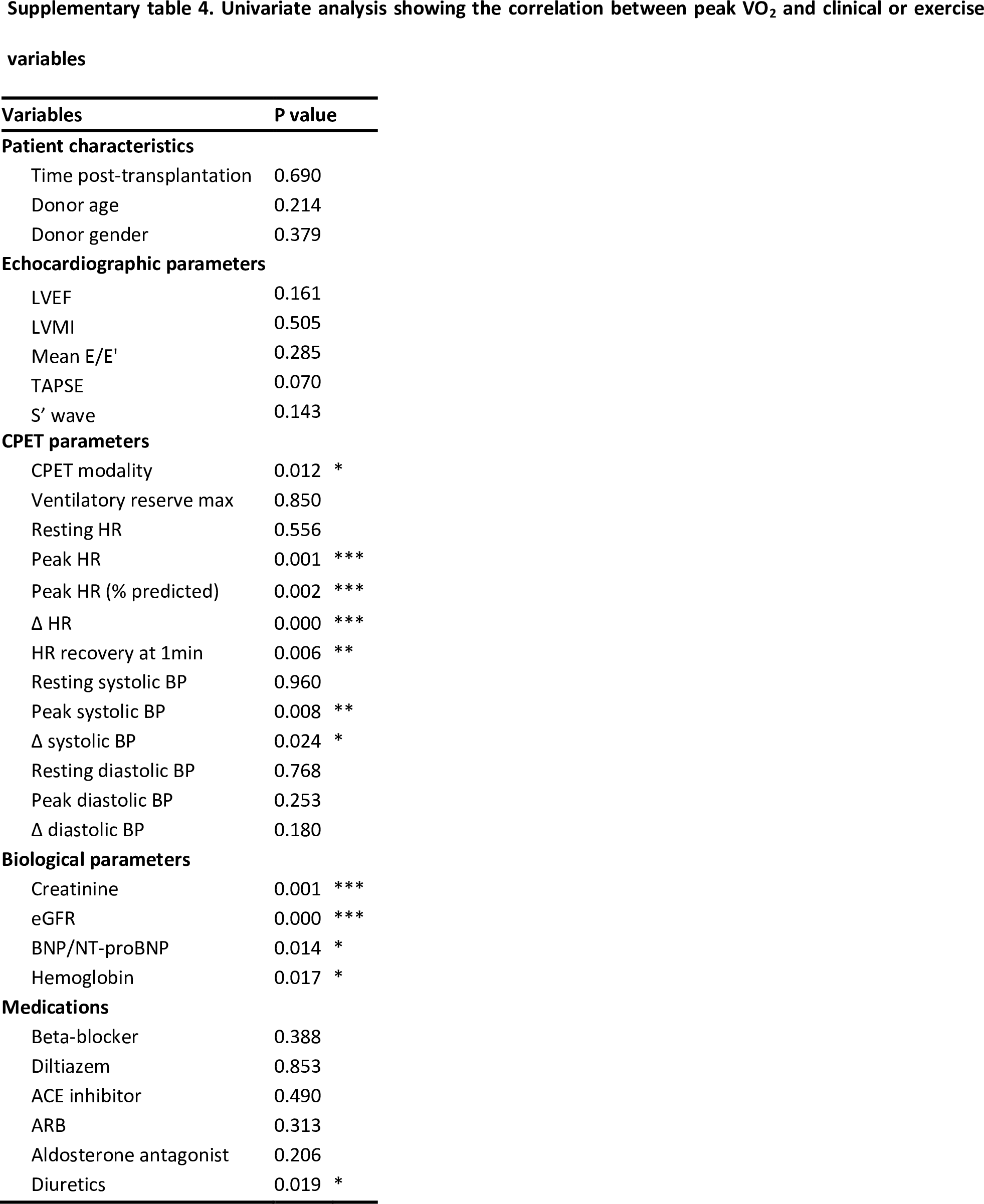

Supplement: S4 Table — LVEF = left ventricular ejection fraction, LVMI = left ventricular mass index, TAPSE = tricuspid annular plane systolic excursion; HR = heart rate; ΔHR = Peak HR—resting HR; BP = Blood Pressure; ΔBP = Peak BP- resting BP; eGFR = estimated glomerular filtration rate; BNP = brain natriuretic peptide; NT-proBNP = N-terminal pro-brain natriuretic peptide; ACE-I = angiotensin converting enzyme-inhibitor; ARB = angiotensin II receptor blocker type 1. (TIF) [file pone.0163475.s005.tif]
